# Case report: Placental chorioadenoma in a primiparous pug dog

**DOI:** 10.3389/fvets.2024.1505470

**Published:** 2024-11-27

**Authors:** Orsolya Balogh, Santiago Diab, Acadia Parker, Gabriela C. de Sousa, Julie T. Cecere, Samantha J. McCarter, Dan Phillip Sponenberg

**Affiliations:** ^1^Department of Small Animal Clinical Sciences, Virginia-Maryland College of Veterinary Medicine, Blacksburg, VA, United States; ^2^Department of Biomedical Sciences and Pathobiology, Virginia-Maryland College of Veterinary Medicine, Blacksburg, VA, United States; ^3^Virginia-Maryland College of Veterinary Medicine, Blacksburg, VA, United States

**Keywords:** canine, placenta, mass, neoplasm, chorioadenoma, bitch, puppy

## Abstract

A single 1.7 cm × 1.2 cm × 1 cm focal, raised, smooth, round, pink to flesh-colored mass protruding from the chorioallantois of the zonary placenta was found during Cesarean section in a primiparous pug bitch. Microscopically, the non-encapsulated, non-infiltrative, exophytic mass originated from the chorioallantoic stroma overlying the labyrinth and was composed of many arborizing fronds generally lined by 1 to 2 layers of cuboidal, columnar and occasionally polygonal, large trophoblastic cells, but frequently by a larger number of cells that occasionally piled up to form small nests or nodules. The gross and microscopic characteristics of this mass were compatible with a benign neoplastic process arising in the chorioallantois and involving placental trophoblasts, hence the diagnosis of a placental chorioadenoma. Five of the six newborn puppies were viable and of normal birthweight, while one puppy, which had lower birthweight than the others, could not be resuscitated and was humanely euthanized. Although placental tumors in dogs are very rare, this case is compelling evidence in the argument for routine inspection of canine placentas to identify potentially deleterious macro- or microscopic conditions that may be linked to negative fetal outcomes.

## Introduction

The definitive canine placenta is classified as chorioallantoic, zonary (complete girdle with marginal hematomas at the border), lamellar, endotheliochorial, and deciduate. It is distinct from the placentas of humans, primates, equids or ruminants. This placental type is shared amongst carnivores and is more invasive than the placentas of other domestic animal species such as the epitheliochorial placenta of the horse and pig, or the synepitheliochorial placenta of ruminants, allowing for efficient exchange of maternal gas and nutrients (1–4).

Histologically, the placentation site in the bitch consists of the placental labyrinth transitioning through the junctional zone into the deeper layers of the endometrium (the glandular zone, connective tissue layer, deep uterine glands), and finally into the myometrium (5). At the time of implantation, morphological signs of decidualization are present in the uterine subepithelial stroma and invasion of trophoblasts from the chorion begins. The chorion becomes vascularized when the allantois attaches to the chorion. With the continued invasion of trophoblast cells forming the chorioallantoic villi, these cytotrophoblasts reach the maternal capillaries giving rise to the definitive endotheliochorial placenta. Syncytial trophoblasts cells sit adjacent to and surround maternal blood vessels and decidual cells. At the feto-maternal interface, fetal projections interdigitate with maternal projections, giving rise to a lamellar organization of the placental labyrinth (3, 5).

Placental masses or tumors in canines are rare. A single case has been reported in a four-year-old Bull mastiff bitch that had a single placental mass in one of the six placentas. The affected placenta was associated with a viable offspring, which had significant growth retardation. Histologically, the structure of the mass resembled that of the labyrinth of the placenta, and based on its characteristics was considered benign and diagnosed as a trophoblastic hamartoma, which is a non-neoplastic entity (6).

Across species, a thought-provoking crossroad exists between placental and neoplastic biology. It has been suggested that the placenta in some species has evolved as a “highly invasive tumor-like organ to derive oxygen and nutrients for the fetus and exchange waste products” (7). The inherently proliferative and invasive nature of trophoblast cells of the human hemochorial placenta have therefore been compared to cancer cells, with which they share several attributes (7). Tumors arising from the placenta can be divided into trophoblastic and non-trophoblastic in origin. In women, the term gestational trophoblastic diseases include tumors of trophoblastic origin such as choriocarcinomas, epithelioid trophoblastic tumors, and placental site trophoblastic tumors, tumor-like conditions such as placental site nodule and exaggerated placental site reaction, and hydatidiform moles (complete, partial, invasive) (8–10). Complete and partial moles are considered premalignant, as they carry the risk of progressing to gestational trophoblastic neoplasia (11). Invasive moles (chorioadenoma destruens) are characterized by myometrial and/or vascular invasion without intervening the decidualized endometrium, and occur in a higher number of complete moles than partial moles (8, 12). Recently, persistent moles, invasive moles, and metastatic moles have been included under the term gestational trophoblastic neoplasia due to the clinically aggressive nature of these lesions (8). Chorioangiomas are the most common non-trophoblastic tumor-like lesions and primarily represent defective capillary or capillary-cavernous blood vessel formation within the stroma resulting in enlarged placental villi. Proliferation rates of the syncytiotrophoblast, fibroblast and endothelial cell components within chorioangiomas may also be increased. Chorioangiomas have been compared to benign hamartomas, although clinical pregnancy complications may occur despite their histologically generally benign nature (13–15).

The literature on human cases of placental tumors is considerable, likely due to the regular inspection of the placenta and the high number of human births. In non-human species, it is unclear whether tumors within the placenta are indeed rare or just poorly documented. Especially in polytocous domestic animal species, inspection of the placentas is not routinely done. A few case reports of tumors of the placenta associated with concurrent gestation have been documented in non-human species. Moles have been described in cows with concurrent normal twin fetus (16, 17), and a stem cell tumor in a bongo (18). In horses, where detailed inspection of the placenta is routine, there have only been two reports of placental tumors; one was a teratoma (19) and the other a mixed germ cell tumor that metastasized to the foal (20). Placental neoplasms involving the reproductive tract in non-pregnant females have also been described, for example, an epithelioid trophoblastic tumor in a non-human primate (21), a choriocarcinoma-like tumor in a maiden pot-bellied pig (22), a choriocarcinoma in a guinea pig (23), or a mole in an embryo donor cow without the presence of a normal fetus (24).

This paper describes the gross and histological characteristics of a placental chorioadenoma found during Cesarean section in a two-year-old primiparous pug bitch and discusses the occurrence and relevance of placental tumors in non-human species.

## Case description

A two-year-old primiparous female intact pug initially presented to the Virginia Tech Veterinary Teaching Hospital's Theriogenology service for gestational aging via ultrasound in preparation for a planned Cesarean section. According to the owner, the bitch was mated accidentally to the housemate male pug with a tie observed approximately 48 days before; it was unclear whether any mating prior to that occurred. Radiographs performed a few days earlier by the referring veterinarian indicated a gravid uterus with at least five fetuses present.

The bitch's physical exam was unremarkable, and abdominal ultrasound showed appropriate heart rates in all fetuses (206–216 bpm), minimal corticomedullary definition of the fetal kidneys, and minimal layering of the fetal gastrointestinal tract in the absence of intestinal motility. Fetal biparietal diameter measurements estimated the date of parturition in 4.5 ± 2 days. No obvious fetal, placental or uterine abnormalities were noted. Ultrasonography was repeated 3 days later and indicated normal fetal vitality (heartrates 216–225 bpm), improved distinction of fetal renal corticomedullary definition, and intermittent fetal gastrointestinal peristalsis. Fetal biparietal measurements estimated parturition in 2.6 ± 2 days, and serum progesterone concentration was 3.78 ng/mL. The bitch returned the next morning after she had a temperature drop (36.6 °C) and started to show intermittent nesting and restlessness indicative of 1st stage labor. After confirming fetal viability (heart rates 183–206 bpm), distinct fetal renal corticomedullary definition and continuous gastrointestinal peristalsis on ultrasound exam, and a serum progesterone of 1.49 ng/mL, a Cesarean section was performed.

Blood serum chemistry and a complete blood count at that time showed clinically unremarkable changes consistent with late term pregnancy (mild regenerative anemia, mild mature neutrophilia, slightly low plasma creatinine, total calcium and increased glucose). Two male and four female puppies and their six associated placentas were removed from the uterus during surgery. Shortly after delivery, the smallest female puppy (birthweight 108 g) went into significant respiratory distress. Active cardiopulmonary resuscitation and medical intervention were pursued with no improvement to its condition for which the puppy was humanely euthanized. The other five puppies weighed between 121 and 135 g and had Apgar scores of 8 out of 10 indicating good vitality and prognosis for survival (25). The six placentas were collected and upon inspection, one of the placentas had a single, focal, mass-like, tissue proliferation. Because the placentas were not matched to the respective newborns upon removal, it is unknown if the placenta with the lesion belonged to the compromised puppy that was ultimately euthanized.

**Figure 1:**
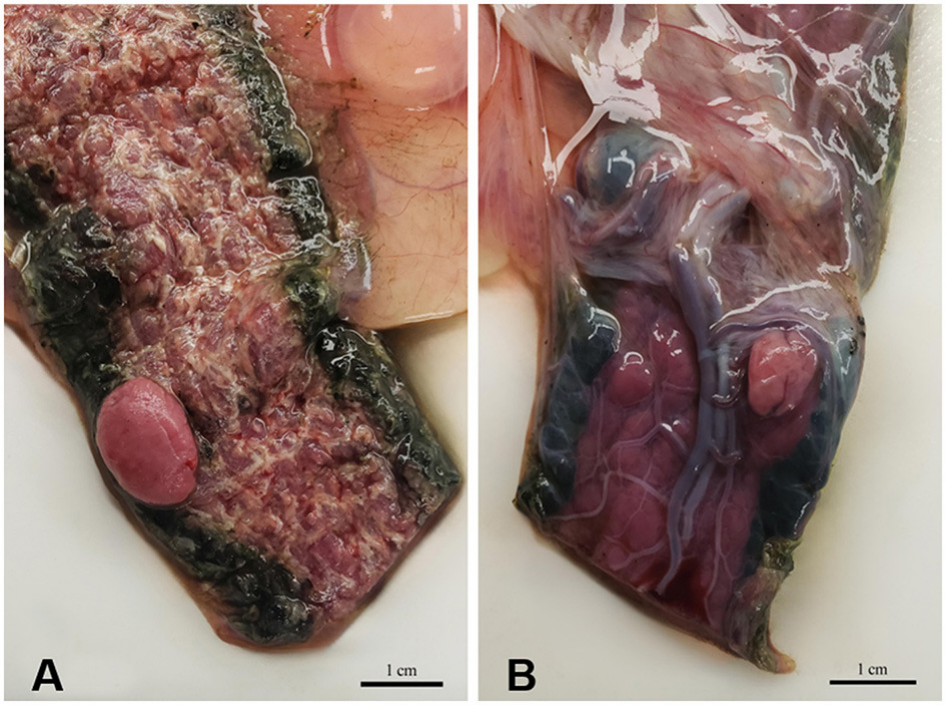
Macroscopic image of the mass protruding **(A)** from the chorionic (external) surface and **(B)** from the allantoic (internal) surface of the zonary placenta.

**Figure 2:**
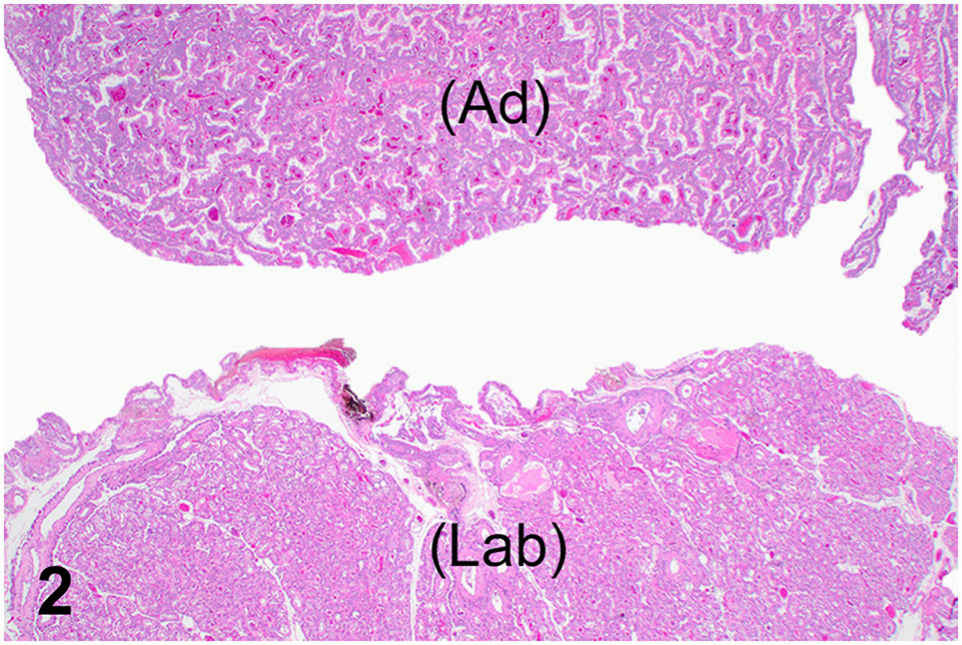
Histology, hematoxylin and eosin stain. Normal placental labyrinth (Lab) and spatial relationship with the non-invasive, exophytic, unencapsulated chorioadenoma (Ad).

**Figure 3:**
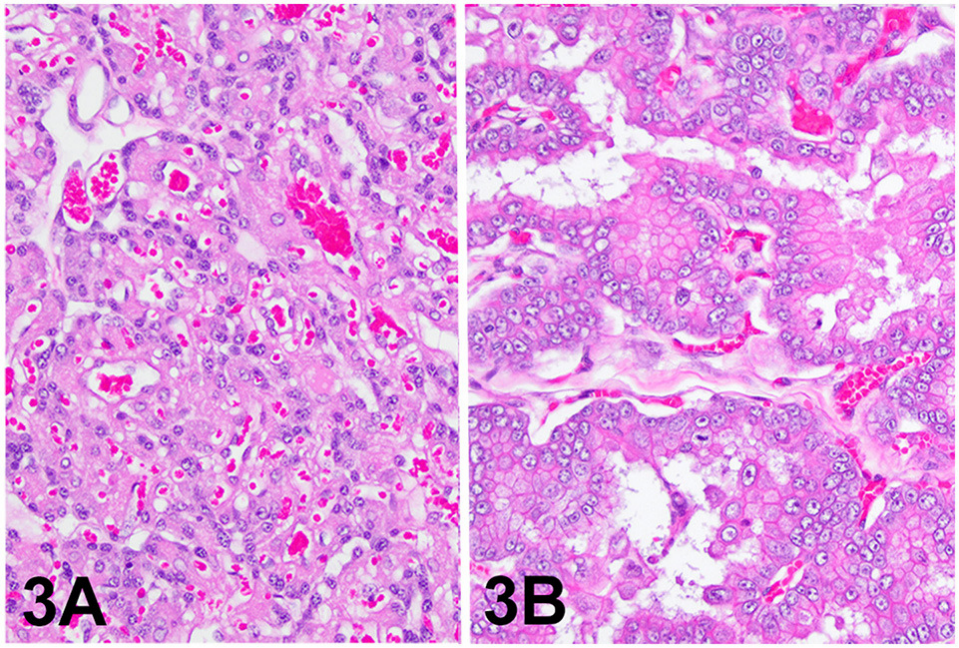
Histology, hematoxylin and eosin stain. **(A)** Placental labyrinth, 200x. **(B)** Chorioadenoma, 200x.

**Figure 4:**
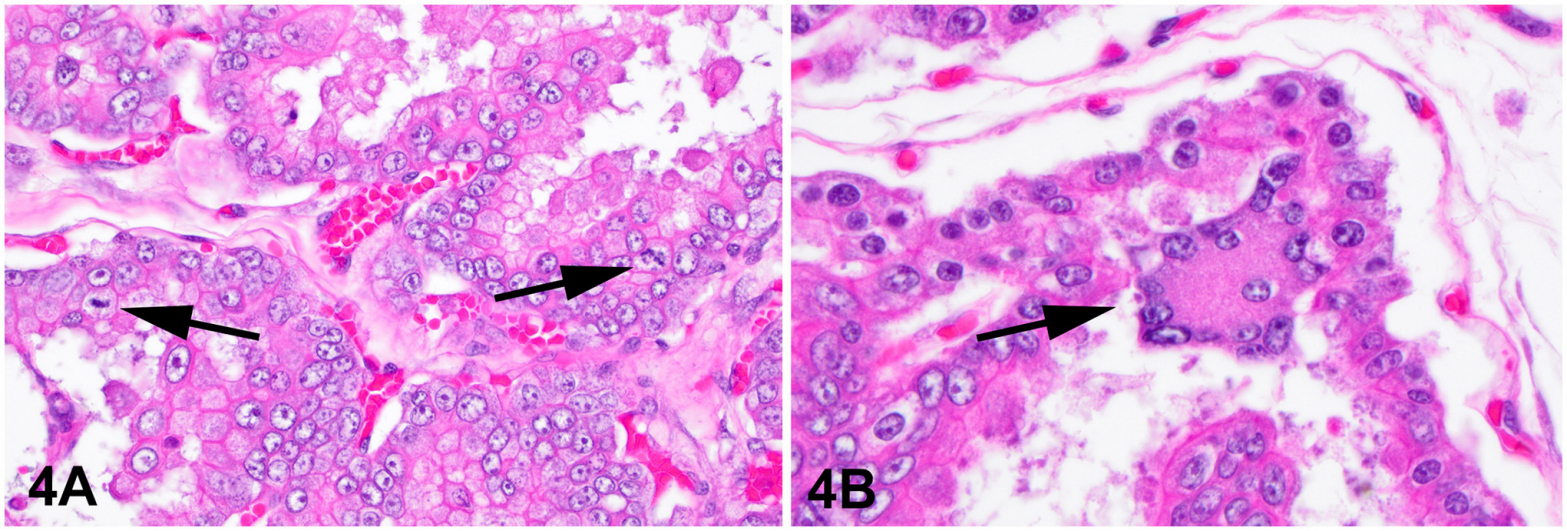
Histology, hematoxylin and eosin stain. Chorioadenoma, 400x. **(A)** Neoplastic trophoblasts pile up to several layers and show frequent mitoses (arrows). **(B)** Trophoblastic syncytia (arrow).

The lesion on the placenta was a single 1.7 cm × 1.2 cm × 1 cm focal, raised, smooth, round, pink to flesh-colored mass at the edge of the zonary placenta in close proximity to the marginal hematoma (Figure 1). The mass protruded with greater proliferation on the chorionic (external) surface of the placenta while also extending to the allantoic (internal) surface (Figure 1). The entire placenta was submitted for histopathology. The tissue was fixed in 10 % neutral phosphate buffered formalin and processed routinely. Standard 4 um sections of tissue were stained with hematoxylin and eosin (H&E) for histological evaluation. Microscopically, the mass originated from the chorio-allantoic stroma overlying the labyrinth (Figures 2, 3) as an exophytic, unencapsulated, non-infiltrative neoplasm composed of many arborizing fronds lined by cuboidal, columnar and occasionally polygonal, large trophoblastic cells separated by a thin band of highly vascular mesenchymal stroma. Fronds were generally lined by 1 or 2 layers of trophoblastic cells but frequently by a larger number of cells that occasionally piled up to form small nests or nodules and not interdigitating with maternal components. The cells had abundant, variably distinct, eosinophilic cytoplasm. Nuclei were round to oval, large, often basilar with occasional loss of polarity, had finely stippled chromatin and 1–3 prominent nucleoli. Anisocytosis and anisokaryosis were moderate. There were 14 mitoses per 10 400x power fields (2.37 mm^2^) (Figure 4A). Additional features included multifocal trophoblast cell sloughing, megalocytosis with karyomegaly, frequent bi- or multinucleation and syncytia formation (Figure 4B). No underlying condition leading to hyperplastic stimulation was evident, such as infection, inflammation, or other changes. The gross and histological characteristics of the mass are compatible with a diagnosis of chorionic placental chorioadenoma.

## Discussion

The proliferative mass presented in this report had features consistent with a benign neoplastic process arising in the chorioallantois and involving placental trophoblasts, namely a placental chorioadenoma. Histological features suggestive of neoplasia include the focal exophytic growth, trophoblastic cell piling, high mitotic rate, moderate cellular pleomorphism, prominent single or multiple nucleoli and megalocytosis. Proliferative lesions can be hypertrophic, hyperplastic, or neoplastic. Although grossly hypertrophy, hyperplasia and neoplasia can look similar, their differences lie in the underlying initiation factors, the type of tissue involved and the microscopic characteristics of the cells and tissue. Hypertrophic and hyperplastic changes are a response to some specific stimulation external to the affected cells and tissues. In some cases, the stimulus is not obvious, but it remains true that these proliferative changes are controlled and not autonomous. In contrast, neoplastic changes (whether benign or malignant) are autonomous and not related to stimulation external to the affected cells. In this case, the lack of any evidence of a stimulus that could have led to focal hyperplasia, the presence of only fetal tissue and no fetal-maternal structures, as in the normal canine placenta, within the mass further support that this is a neoplastic, rather than a hyperplastic process. Gestational neoplastic transformation of trophoblastic cells is a known entity in humans, however, in veterinary medicine, the only trophoblastic neoplasms that have been described were mola hydatidosa in cows (16, 17). A proliferative placental mass of trophoblastic origin was described in a bitch with some similarities to the mass presented here (6). That study, however, reported the mass as a placental hamartoma which, by definition, is a non-neoplastic transformation. To date, other case reports on gestational placental neoplasms in domestic animal species described a stem cell tumor (18), a teratoma (19), and a mixed germ cell tumor (20).

In the report of Cushings et al. (6), the neonate puppy associated with the placental hamartoma was approximately one-third of the birthweight of its littermates. The authors suggested that the mass, which measured 4 cm × 4 cm × 2cm, may have had a direct compressive effect on placental perfusion, resulting in fetal growth retardation. This puppy was however viable, despite the bitch's pre-term labor that had to be medically managed until the fetuses were mature to be safely delivered via Cesarean section. In contrast to the complicated clinical presentation and reproductive history of the dam in that report (6), the bitch in the present case had an uneventful pregnancy. Unfortunately, we could not determine with certainty whether the placental chorioadenoma had clinical association with the smallest puppy that was euthanized, because the placentas were not matched with the individual puppies. The placental chorioadenoma in this case was near large vessels of the chorioallantois and it could have caused some degree of compression to the vascular supply of the placenta and to the umbilical cord due to its expansile nature, potentially leading to growth retardation and neonatal demise. In the hypothetical scenario that the placental chorioadenoma in this case matched the placenta of the smallest puppy from this litter, the smaller size of the placental mass (1.7 cm × 1.2 cm × 1 cm) compared to the one reported previously (6) may explain why the birthweight of this puppy was not much lower compared to the other neonates.

Any derangements affecting placental integrity (e.g., infection, inflammation, vascular pathology, inadequate placental size or development) can interfere with fetal development (26) and cause growth retardation. For example, in horses where the placenta is closely inspected and weighed following parturition, placental weight and foal weight are positively associated (27), with the normal placenta in Thoroughbreds being approximately 11 % of the foal's birth weight (28). In the dog, placental weight to birth weight ratio ranged between 11.0 and 22.3 % in healthy puppies of various breeds with significant, high correlations between the two, i.e., each 1 g increase in placental weight accounted for 6.77 g increase in puppy birth weight (29). In toy and small breed dogs, puppy birth weight and placental weight ratio was 7.85 % and 6.89 %, respectively (30), while Sarli et al. (31) found ratios between 7.2 and 33.3 % depending on litter size. In dogs, it is impossible to match all or most of the placentas to the puppies born in cases of natural birth or even at Cesarean section delivery, complicated with the fact that bitches tend to consume their placentas. Inspection of the canine placenta may be an important clue in determining factors linked to fatal outcomes (31). Therefore, in order to better understand canine placental abnormalities and their relationship to puppy birthweight and vitality, routine inspection of placentas should be performed whenever possible.

## Concluding remarks

This is the first report of a placental chorioadenoma found in a term pregnant bitch. The presence of this mass may have caused the demise of the newborn puppy by disrupting placental function. Placental masses in the bitch are rare and may be underreported due to the lack of routine inspection of canine placentas. This underscores the need for more studies on macro- and microscopic assessment of placentas in dogs, especially in cases of compromised or low birthweight neonates.

## Data Availability

The raw data supporting the conclusions of this article will be made available by the authors, without undue reservation.
